# A novel animal model for neuroinflammation and white matter degeneration

**DOI:** 10.7717/peerj.3905

**Published:** 2017-10-31

**Authors:** Baohu Ji, Kerin Higa, Virawudh Soontornniyomkij, Atsushi Miyanohara, Xianjin Zhou

**Affiliations:** 1 Department of Psychiatry, University of California, San Diego, La Jolla, CA, United States of America; 2 Department of Anesthesiology, University of California, San Diego, La Jolla, CA, United States of America; 3 Research Service, VA San Diego Healthcare System, San Diego, CA, USA

**Keywords:** shRNA, Drd1, Microglial activation, Innate immunity, Neuroinflammation, White matter degeneration

## Abstract

Small interference RNA has been widely used to suppress gene expression. Three different short hairpin RNAs (shRNAs) against dopamine D1 receptor (Drd1), driven by mouse U6 promoter in self-complementary AAV8 vector (scAAV8), were used to silence mouse striatal Drd1 expression. Transduction of mouse striatum with all three scAAV8-D1shRNA viruses, but not the control scAAV8 virus, causes extensive neuroinflammation, demyelination, and axon degeneration. RNA interference is known to be coupled to the innate immune system as a host cell defense against virus infection. Activation of the innate immune system may play a causal role in the development of neuroinflammation and white matter degeneration, providing a novel animal model for multiple sclerosis (MS) and other neuroinflammatory diseases.

## Introduction

RNA interference has been widely used to suppress gene expression in mammalian cells (Reynolds et al., 2006) and *in vivo* (Davidson & Harper, 2005; Harper & Davidson, 2005; Harper et al., 2005). However, both long and short interference RNAs, recognized as viral double-stranded RNAs (dsRNAs) by host cells (Umbach & Cullen, 2009), activate the innate immune system to induce expression of pro-inflammatory cytokines via either sequence-independent or -dependent pathways (Hornung et al., 2005; Judge et al., 2005; Sledz et al., 2003). Short hairpin RNA (shRNA) was suggested to have less immunogenicity since it is processed by endogenous microRNA pathway (Rao et al., 2009; Robbins et al., 2006). However, overexpression of nonspecific shRNAs using AAV1/2 virus activated microglial cells in mouse striatum (McBride et al., 2008), suggesting that shRNA immunogenicity rather than shRNA silencing effects play a causal role. Neither demyelination nor axon degeneration were reported in the study.

Different AAV viral vectors and serotypes have been developed to express shRNA in the central nervous system (CNS). Among these vectors, self-complementary adeno-associated virus (scAAV) with a double-stranded DNA bypasses the rate-limiting step of second DNA strand synthesis in traditional AAV virus (single-stranded DNA) and thereby transduces target tissue much more efficiently (Fu et al., 2003; McCarty et al., 2003; McCarty, Monahan & Samulski, 2001). Among different AAV serotypes, AAV8 was reported to be more efficient to transduce mouse brains than AAV1 and AAV2 (Broekman et al., 2006).

Striatal dopamine D1 receptor (Drd1) plays an important role in a number of neurological diseases including Parkinson disease, Huntington disease, addiction, depression, and schizophrenia (Gantois et al., 2007; Gerfen, 1992; Knutson & Gibbs, 2007; Le Foll et al., 2009; Nagatsua & Sawadab, 2009; Zhu et al., 2005). Dopamine is a principal neurotransmitter in the CNS, and is also an important modulator in regulation of neuroinflammation (Levite, 2016). To investigate Drd1 functions, we constructed recombinant scAAV8 virus to express short-hairpin RNAs (shRNA) to specifically silence Drd1 expression in mouse striatum. We found that all scAAV8-D1shRNA viruses cause extensive neuroinflammation, demyelination, and axon degeneration in mouse brain.

## Materials and Methods

### D1shRNA target sequences

The target sequence of the first Drd1 shRNA (shRNA1) was chosen according to *in vitro* studies (Isacson et al., 2003). The other two D1shRNAs were designed by *in silico* selection. Two web based siRNA selection programs were used for the design of shRNAs (MIT: http://jura.wi.mit.edu/bioc/siRNAext/; and U-Tokyo: http://alps3.gi.k.u-tokyo.ac.jp/ yamada/sidirect2/index.php?type=fc). Analysis of potential “off-target” effects for each D1shRNA was conducted at the MIT web site (http://jura.wi.mit.edu/bioc/siRNAext/). The second D1shRNA2 (shRNA2) target region was chosen from 1,024 to 1,046 of Drd1 mRNA, 5′ GGCCCTTGGATGGCAATTTTACT 3′. The third D1shRNA3 (shRNA3) target region was chosen from 2,778 to 2,800 of Drd1 mRNA, 5′ AAGAGCATATGCCACTTTGTATT 3′. These shRNAs were selected due to their relatively low potential off-target effects in mouse genome, particularly at position 2 to 13 of the guide strand. The potential off-target effects for each shRNA were also analyzed *in silico*, and no common unintended genes were found.

### Construction of recombinant scAAV8 virus

To minimize potential toxicity of fluorescence marker protein, EGFP was replaced with humanized *Renilla* green fluorescence protein (hrGFP). The CMV-hrGFP cassette was released from pAAV-CMV-hrGFP (kindly provided by Dr. Beverly Davidson) by double digestion with EcoR I and Xba I enzymes, and was used to replace CMV-EGFP cassette in scAAV vector pVm-CMV-EGFP digested with EcoR I and Xho I enzymes. The resultant scAAV-hrGFP vector (control) was used to clone all shRNAs at the EcoR I site. Short-hairpin RNA cassette was driven by mouse U6 promoter with a loop sequence (5′ CTTCCTGTCA 3′). Three different D1shRNAs (shRNA1, shRNA2, shRNA3) targeting different *Drd1* regions were cloned under the control of mouse U6 promoter. AAV8 help plasmid (kindly provided by University of Pennsylvania) was used for the generation of recombinant scAAV8 virus. The titers of the four scAAV8 virus were about 2 × 10^12^.

### Mouse strain and surgery

Male C57BL/6J adult mice were purchased from Jackson Laboratory. Mice were housed in a climate-controlled animal colony with a reversed day/night cycle. Food (Harlan Teklab, Madison, WI) and water were available *ad libitum*. After a week of acclimation, 10 to 20 mice were grouped for injection of each scAAV8 viruses (control, shRNA1, shRNA2 and shRNA3). The virus was injected at both dorsal (Bregma +0.86, *M*∕*L* ± 1.65 , *D*∕*V* − 2.45 mm) and ventral striatum (Bregma +0.86, *M*∕*L* ± 1.65 , *D*∕*V* − 3.8 ) of 3-month old C57BL/6J male mice. 1 µl volume of scAAV8 virus was injected slowly (2 min) using stereotaxic delivery at each site. The surgery and other procedures were approved by both the UCSD Animal Care and Use Committee (UCSD Animal Use Protocol: S04190M) and local Veteran’s Administration Hospital (Animal Use Protocol: 04-036 (VAH)) prior to the onset of the experiments. Mice were maintained in American Association for Accreditation of Laboratory Animal Care approved animal facilities. This facility meets all Federal and State requirements for animal care.

### Western blot and Immunohistochemistry

Mice were euthanized with carbon dioxide, and the brains were quickly removed on ice. Both anterior and posterior striatum were dissected from coronal brain sections on ice. Tissues were homogenized using Dounce Homogenizer in 1X Passive Lysis Buffer (Promega, Fitchburg, WI, USA) supplemented with Protease Inhibitor Cocktail (Sigma, St. Louis, MO, USA). Protein concentration was measured with Bradford Assay Kit (Pierce™, Thermo Fisher Scientific, Waltham, MA, USA). 20 µg of total proteins were loaded on SDS-PAGE gel, and blotted to PVDF membrane (Bio-Rad, Pleasanton, CA, USA) after separation with electrophoresis. Mouse monoclonal antibody anti-Drd1 (abcam, Cambridge, UK) and mouse monoclonal antibody anti-NMDAR1 (BD Biosciences, San Jose, CA, USA) were used as primary antibodies. HRP conjugated anti-mouse IgG and HRP conjugated anti-rabbit IgG, were obtained from Amersham and Sigma as the secondary antibodies. The concentration of primary antibodies for Western Blot was generally used as recommended by the manufacturers. Image J was used for quantification. Immunohistochemical staining on coronal brain sections was performed as previously described (Kim et al., 2012). Mouse monoclonal antibody anti-Drd1 (abcam), rabbit anti-Drd1 (Sigma), rabbit anti-Iba-1 (Wako, Osaka, Japan, 019-19741); rabbit anti-MBP (Dako, #A0623), and goat anti-NF-L (C-15, Santa Cruz, #sc-12980) were used as primary antibodies. Mouse brains were analyzed 7 weeks post-injection. In brief, paraffin brain sections were baked, deparaffinized, and rehydrated. After rinsed in distilled water, the sections were submerged in Tris-EDTA Buffer (10 mM Tris Base, 1 mM EDTA Solution, 0.05% Tween 20, pH 9.0), and autoclaved (Tuttnauer, Hauppauge, NY, 2,340 M) for antigen retrieval at 121°C for 20 min. After autoclaving, endogenous peroxidase activity was quenched with incubation of 0.3% hydrogen peroxide in PBS for 30 min at room temperature. The slides were washed and blocked with 2.5% normal horse serum (ImmPRESS, Vector Labs, Burlingame, CA, USA, S-2012). After incubation with the primary antibodies, ImmPRESS peroxidase-micropolymer conjugated horse anti-mouse, anti-rabbit, anti-goat IgG (Vector Labs) antibodies were used as the secondary antibody. Chromogenic reaction was conducted with ImmPACT NovaRED Peroxidase Substrate (Vector Labs, SK-4805) for 5 min with rotational shaking. After staining, the slides were washed and air-dried overnight. Next day, the slides were mounted with Cytoseal 60 mounting medium (Richard-Allan Scientific, San Diego, CA, USA, 8310-16).

**Figure 1:**
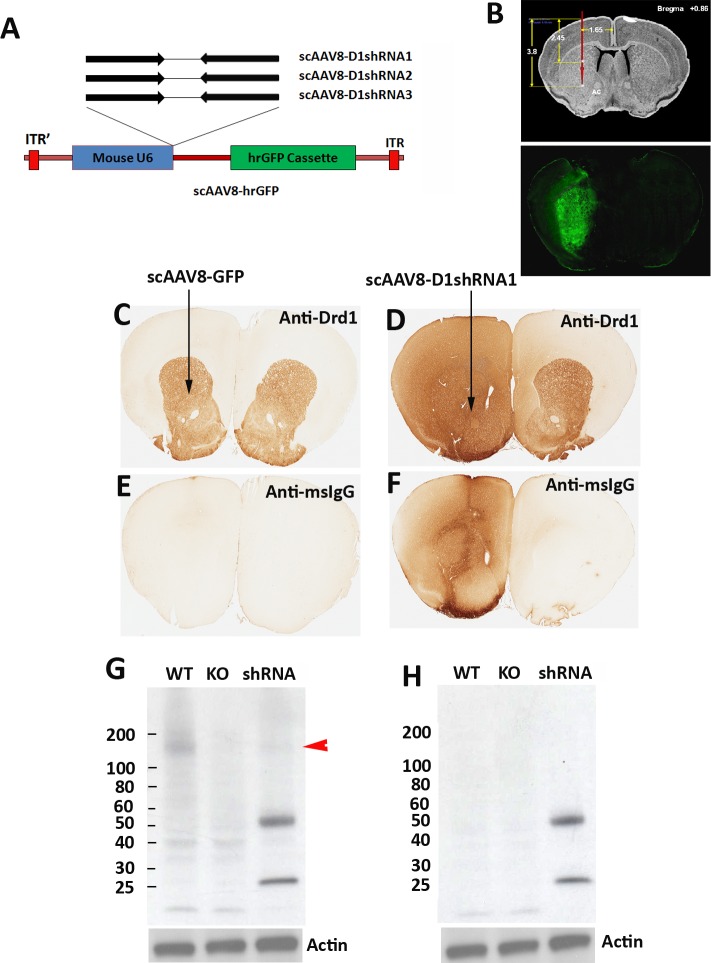
Massive mouse IgG proteins in brain tissue transduced with scAAV8-D1shRNAs. (A) Three shRNAs targeting different sites of mouse Drd1 mRNA were designed to suppress Drd1 expression. All three Drd1 shRNAs were under the control of mouse U6 promoter in scAAV-hrGFP vector. (B) The coordinates for the stereotaxic injection of scAAV8 virus into mouse striatum. Transduction efficiency of scAAV8 was examined by hrGFP expression in mouse striatum 4 weeks after surgery. Immunohistochemical staining of striatal Drd1 protein was conducted using mouse anti-Drd1 antibody in control mice (C) injected with scAAV8-hrGFP virus or mice injected with scAAV8-D1shRNA1 virus (D) 7 weeks post-injection. The virus was injected only into the left hemisphere as indicated by the arrows. ImmPRESS peroxidase-micropolymer conjugated horse anti-mouse IgG secondary antibody (without primary antibodies) was used for immunostaining of the control brain section (E) from mice injected with scAAV8-hrGFP virus and the brain section (F) from mice injected with scAAV8-D1shRNA1 virus. (G) Western blot analysis of Drd1 expression in the striatum of wildtype and Drd1 knockout mice as well as mice injected with scAAV8-D1shRNA1 virus. A putative Drd1 band migrating between 100 to 200 kD (red arrowhead) may be oligomers of Drd1 proteins that are absent in the knockout mice and reduced in the mice injected with scAAV8-D1shRNA1 virus. Mouse IgG heavy (50 kD) and light chains (25 kD) were detected only in mice injected with scAAV8-D1shRNA1 virus. The same set of samples was analyzed in Western blot with the anti-mouse IgG secondary antibody only (H). Massive mouse IgG was confirmed in mouse brain tissue transduced with scAAV8-D1shRNA1 virus.

## Results

### Massive IgG antibodies in transduced brain tissue

**Figure 2:**
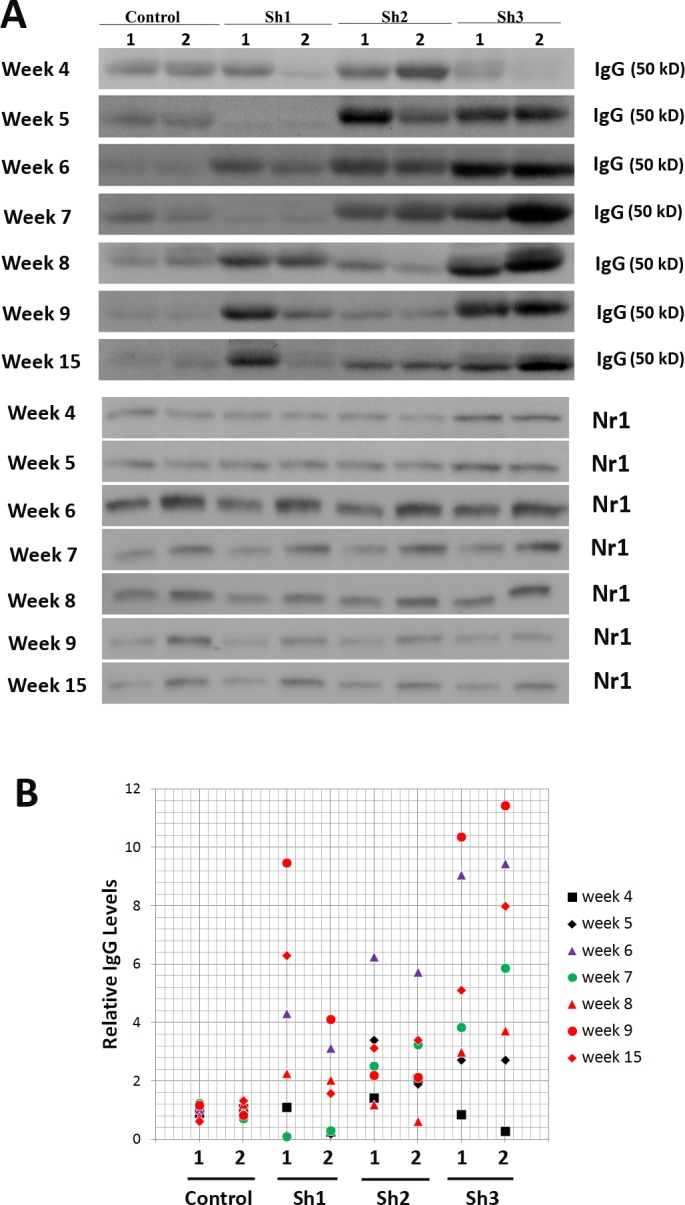
Longitudinal studies of excessive mouse IgG antibodies in mouse striatum transduced with all three scAAV8-D1shRNA virus. (A) Both anterior and posterior striatum were dissected from mice from week 4 to week 15 post-injection. Posterior: 1; anterior: 2. Control = scAAV8-hrGFP, Sh1 = scAAV8-D1shRNA1, Sh2 = scAAV8-D1shRNA2, Sh3 = scAAV8-D1shRNA3. Western blot analysis of mouse IgG was performed with anti-mouse IgG secondary antibody only. Equal amount of proteins were loaded and Nr1 protein was used as an internal control. (B) Image J was used to quantify intensities of mouse IgG bands. To compare IgG signal intensities between different Western blots, IgG intensities were first normalized against the controls within each blot. After normalization by Nr1, IgG levels from the controls and D1shRNAs were compared. Striatum transduced by D1shRNA3 virus had a significantly higher level of IgG (Student’s *t*-test, unequal variance, *p* < 0.01) than striatum transduced with the control hrGFP virus. Many mice injected with either scAAV8-D1shRNA1or scAAV8-D1shRNA2 virus also displayed excessive mouse IgG in their striatum.

To silence Drd1 expression, we designed three different short hairpin RNAs (shRNA) that have lengths ranging from 21 to 23 bp. Both the D1shRNA1 and the D1shRNA2 were designed to targeting the coding sequences of mouse *Drd1* gene, and the D1shRNA3 was selected to targeting the 3′ UTR of the *Drd1* gene (Fig. 1A, Fig. S1A). All three D1shRNAs were driven by mouse U6 promoter in scAAV8 (self-complementary AAV) shuttle vector expressing humanized *Renilla* green fluorescent protein (hrGFP). Recombinant scAAV8-hrGFP virus was generated as a control. Using stereotaxic delivery, each scAAV8 virus was injected only into mouse left striatum, whereas uninjected mouse right striatum served as a control within the same coronal brain section (Fig. 1B). Four weeks after virus injection, hrGFP expression was examined for transduction efficiency. Consecutive cryostat sections (from Bregma 1.86 to −0.86) confirmed that the scAAV8 virus transduced a high volume of mouse striatum (Broekman et al., 2006). To evaluate suppression of Drd1 proteins in striatum, we conducted immunohistochemical staining of paraffin brain sections from both the control hrGFP and the D1shRNA1 mice. Drd1 protein was readily detected in mouse striatum by immunohistochemical staining using mouse anti-Drd1 antibody (Fig. 1C). Surprisingly, the antibody generated extensive “background” across the left brain hemisphere transduced with scAAV8-D1shRNA1, but not in the uninjected right brain hemisphere (Fig. 1D). Adjacent brain sections were then immunostained with anti-mouse IgG secondary antibody only. As expected, the secondary antibody did not detect any signal in mouse brains transduced with scAAV8-hrGFP (Fig. 1E). However, the secondary antibody detected extensive mouse IgG proteins in the left brain hemisphere transduced with scAAV8-D1shRNA1, but not in the uninjected right brain hemisphere (Fig. 1F), suggesting that scAAV8-D1sRNA1 transduction generated massive mouse IgG antibodies in the left brain hemisphere. We performed Western blot to confirm the presence of mouse IgG and Drd1 suppression in mouse striatum (Fig. 1G). As expected, Drd1 protein was reduced (red arrowhead) in the striatum transduced with scAAV8-D1shRNA1, and was absent in the striatum of *Drd1* knockout mice (Fig. 1G). Using rabbit anti-Drd1 antibody to avoid massive mouse IgG “background”, we confirmed reduction of Drd1 proteins with immunnohistochemical analysis in the striatum transduced with scAAV8-D1shRNA1 (Figs. S1B–S1E). Consistent with the finding of massive mouse IgG antibodies in brain tissue by immunohistochemical analysis, we observed mouse IgG heavy (50 kD) and light chains (25 kD) in Western blot analysis using mouse anti-Drd1 antibody (Fig. 1G). Western blot analysis using only anti-mouse IgG secondary antibody confirmed that these two bands were indeed mouse IgG heavy and light chains (Fig. 1H). We were curious about a large amount of mouse IgG antibodies specifically present in the striatum transduced with scAAV8-D1shRNA1 virus, but not in the striatum of either wildtype or *Drd1* knockout mice. To examine whether massive mouse IgG antibodies were also present in mouse striatum transduced by other scAAV8-D1shRNAs, we performed Western blot analyses of mouse striatum transduced with either the control hrGFP or each D1shRNA virus at different post-injection time points (Fig. 2A). Mouse IgG levels were remarkably increased in the striatum of mice injected with all scAAV8-D1shRNAs, particularly scAAV8-D1shRNA3, in comparison with the control mice injected with scAAV8-hrGFP. Longitudinal studies from week 4 to week 15 revealed persistent excessive mouse IgG antibodies in striatum for several months. After normalization against the control in each Western blot, we combined all mouse IgG data (Fig. 2B). Striatum transduced with scAAV8-D1shRNA3 virus had a significantly higher level of IgG (Student’s *t*-test, *p* < 0.01) than the control striatum transduced with scAAV8-hrGFP. Many mice injected with either scAAV8-shRNA1 or scAAV8-shRNA2 virus also displayed excessive mouse IgG antibodies in their striatum. Consistent with the Western blot, we found that almost all mice injected with scAAV8-D1shRNAs displayed IgG background in immunohistochemical staining with anti-mouse IgG antibodies (Table S1). We did not observe differences in IgG staining between the left injected striatum and the right control striatum in scAAV8-hrGFP mice. A low level of striatal IgG detected in scAAV8-hrGFP mice by the Western blot, but not by the immunohistochemical staining, may be caused by different sensitivities of the two techniques and individual mouse variability.

### Extensive microglial activation

**Figure 3:**
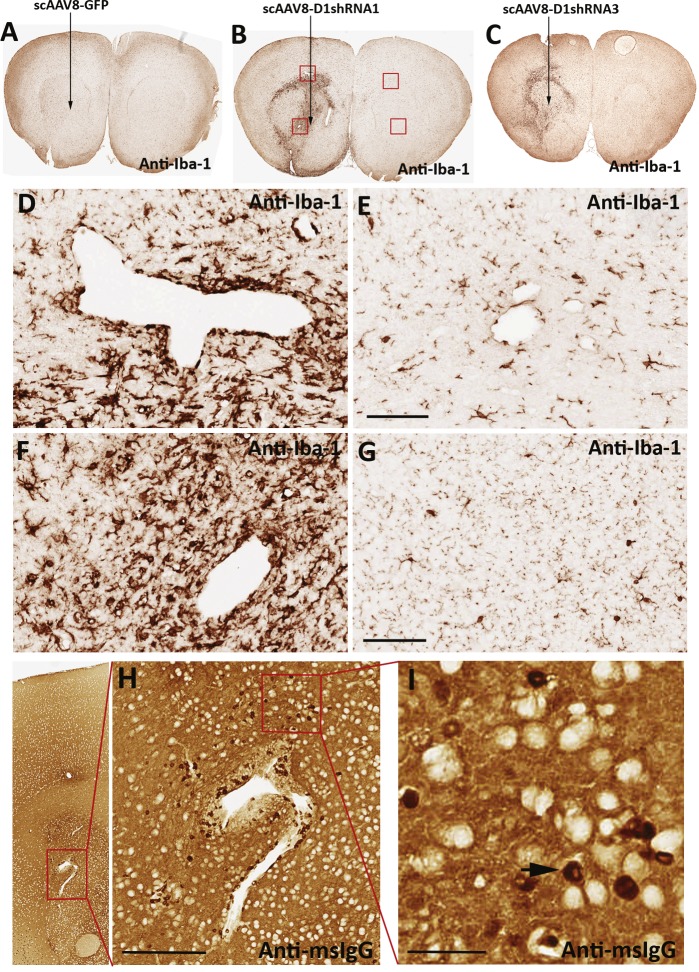
Neuroinflammation and microglial activation. Immunohistochemical staining of the Iba-1 protein, a marker of activated microglia, in mouse striatum transduced with scAAV8-hrGFP (A), scAAV8-D1shRNA1 (B), and scAAV8-D1shRNA3 (C) virus. The virus was injected into the left striatum only (arrow). Activation of microglial cells, as shown by extensive cell proliferation and hypertrophy, was observed in the left striatum injected with either scAAV8-D1shRNA1 or scAAV8-D1shRNA3, but not with scAAV8-hrGFP virus. Blood capillaries surrounded by activated microglial cells were markedly swollen in the left striatum transduced with scAAV8-D1shRNA1 virus (D, F) compared with the control right striatum (E, G) on the same brain section. Scale bar: 100 µm. No visible neuroinflammation or swelling of blood capillaries were observed in the left striatum transduced with the control scAAV8-hrGFP virus. (H) Paraffin sections of scAAV8-D1shRNA mouse brains were immunostained with anti-mouse IgG antibody only. Scale bar: 200 µm. Numerous IgG-positive cells scattered around a swollen blood capillary were putative peripheral immune cells (I, black arrow) infiltrated into the inflammatory brain tissue. Scale bar: 40 µm.

Excessive mouse IgG antibodies in striatum indicate neuroinflammation. We examined microglial activation using immunohistochemical staining of the Iba-1 protein. We found that microglial cells were extensively over-proliferated and hypertrophied in the striatum transduced with scAAV8-D1shRNAs, but not scAAV8-hrGFP virus (Figs. 3A–3C). These data suggested that D1shRNA over-expression, but not the scAAV8 virus itself, causes neuroinflammation. Consistent with neuroinflammation, blood capillaries were swollen and surrounded by activated microglial cells (Figs. 3D–3G). Peripheral immune cells intensively labeled with IgG infiltrated into the inflammatory brain tissue surrounding a swollen capillary, indicating a breakdown of blood brain barrier (Figs. 3H and 3I). Such an intensive neuroinflammation is likely responsible for excessive mouse IgG antibodies in the striatum transduced with scAAV8-D1shRNAs. These IgG antibodies may come from either leakage of blood brain barriers or be produced by infiltrated B cells in the inflammatory brain tissues (Pachner, Brady & Narayan, 2007; Pachner, Li & Lagunoff, 2011; Weber, Hemmer & Cepok, 2011). Persistent excessive IgG antibodies indicate chronic inflammation. We examined the extent of neuroinflammation in individual mice transduced with different scAAV8-D1shRNAs using anti-Iba-1 immunostaining as a marker (Table 1). Massive activation of microglial cells in Figs. 3B and 3C was arbitrarily ranked as “+ +  +”, whereas localized mild microglial activation in Fig. S2 was ranked as “+”. Mice were examined blind to scAAV8 virus genotypes. After analyzing 34 individual mice injected with either scAAV8-hrGFP or scAAV8-D1shRNAs, we did not find microglial activation in any control mouse brain (*n* = 6) transduced with scAAV8-hrGFP virus. Almost all mouse brains transduced with scAAV8-D1shRNA virus displayed microglial activation (Table 1).

**Table 1:** Microglial activation in individual mice.

	Localized aggregation of activated microglial cells (Iba-1 staining)					
	Negative	+ + +	+ +	+	No. of mice	Microglial activation (%)
scAAV8-GFP	6	0	0	0	6	0
scAAV8-D1shRNA1	0	6	3	1	10	100
scAAV8-D1shRNA2	1	2	4	3	10	90
scAAV8-D1shRNA3	1	2	3	2	8	88

### Demyelination and axon degeneration

**Figure 4:**
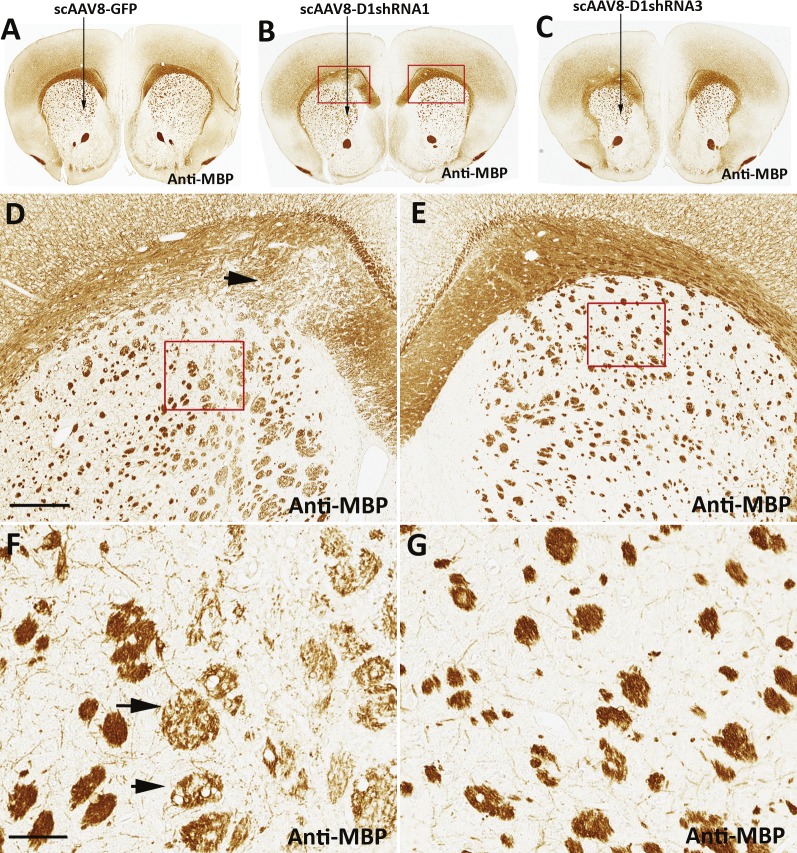
Reduction of MBP in disorganized white matter. Immunohistochemical staining of MBP was conducted in brain sections of mice injected with scAAV8-hrGFP (A), scAAV8-D1shRNA1 (B), or scAAV8-D1shRNA3 (C) virus. The virus was injected only in the left striatum, and the analysis was performed 7 weeks post-injection. MBP staining was reduced in the swollen and disorganized corpus callosum (D, black arrow) of mice injected with scAAV8-D1shRNA1 in comparison with the normal right corpus callosum (E). Scale bar: 200 µm. Reduction of MBP staining was also observed in striatal white matter tracts that were swollen and blebbed (black arrows) in the left striatum transduced with scAAV8-D1shRNA1 (F) in comparison with normal white matter tracts in the control right striatum (G). Scale bar: 60 µm.

**Figure 5:**
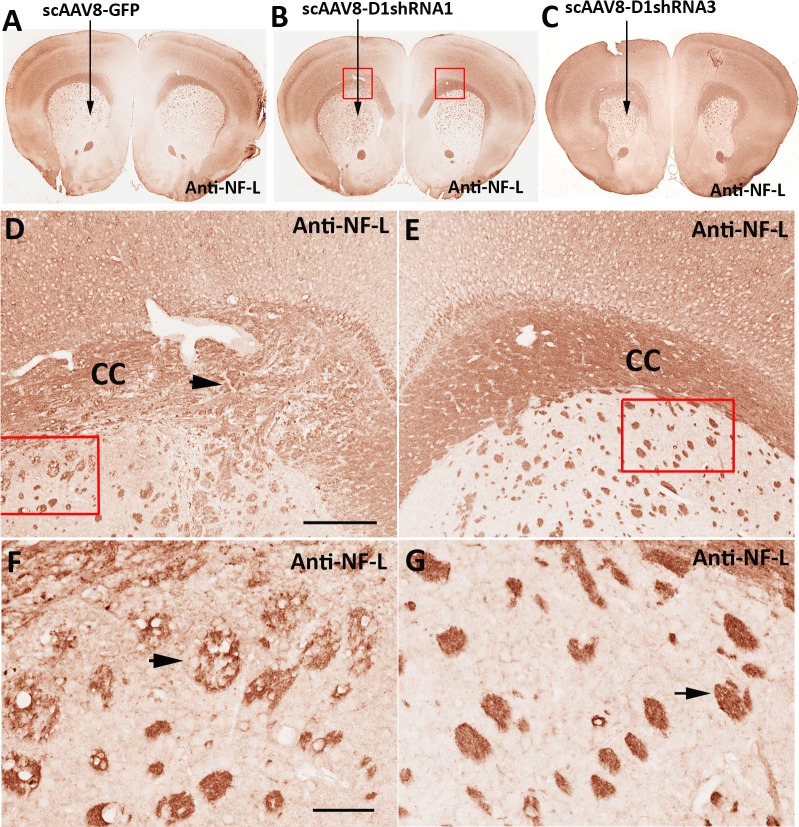
Reduction of neurofilaments inside white matter. We immunostained NF-L proteins on paraffin sections that are consecutive to the sections used in the previous immunostaining of Iba-1 (Figs. 3A–3C) to demonstrate a co-localization between microglial activation and axon degeneration. Immunohistochemical staining of NF-L was conducted in brain sections of mice injected with scAAV8-hrGFP (A), scAAV8-D1shRNA1 (B), or scAAV8-D1shRNA3 (C) virus. Activated microglial cells were preferentially concentrated on corpus callosum. High magnification of immunostaining of neurofilament light chain (NF-L) in mice injected with scAAV8-D1shRNA1 demonstrated that neurofilaments were reduced in the swollen and disorganized corpus callosum (D, black arrow) on the left side in comparison with the normal corpus callosum on the right side (E). Scale bar: 200 µm. Striatal white matter tracts were also swollen and blebbed (black arrow) in the left striatum transduced with scAAV8-D1shRNA1 (F) in comparison with normal white matter tracts (black arrow) in the control right striatum (G). Scale bar: 60 µm.

**Figure 6:**
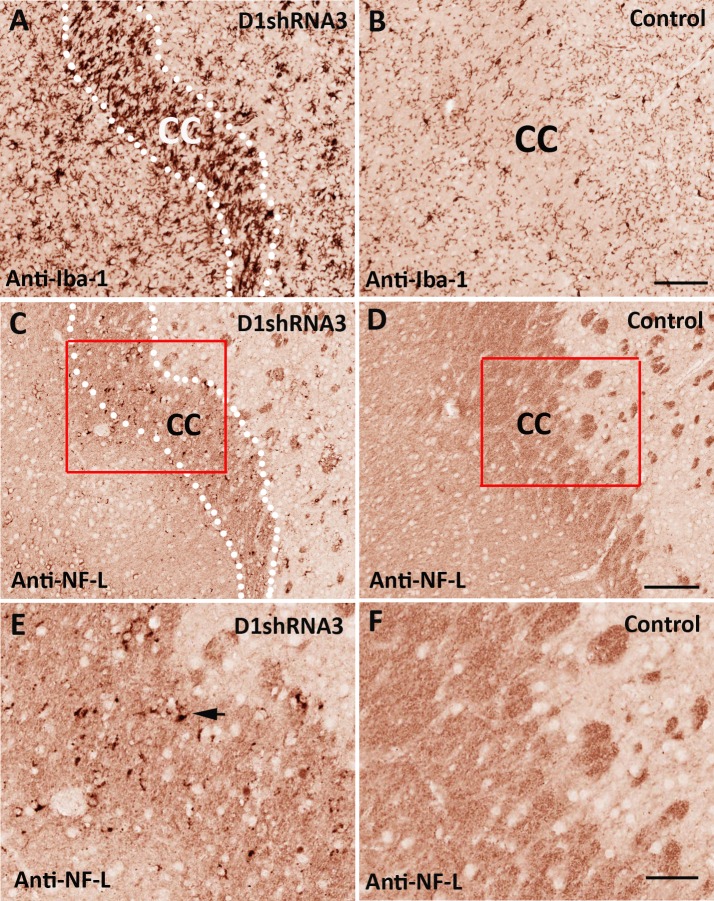
Degeneration of large-caliber axons inside white matter. Adjacent paraffin sections from mice injected with scAAV8-D1shRNA3 virus were immunostained with anti-Iba-1 and anti-NF-L antibodies, respectively. A high density of activated microglial cells (anti-Iba-1 staining) was shown in the left striatum injected with scAAV8-D1shRNA3 virus (A) in comparison with the uninjected control right striatum (B). Scale bar: 120 µm. On an adjacent brain section, decreased overall NF-L staining and various NF-L aggregates were observed on the left corpus callosum transduced with scAAV8-D1shRNA3 virus (C), compared with the staining of the control right corpus callosum (D). Scale bar: 120 µm. Comparison between adjacent brain sections (A and C) showed that activated microglial cells were concentrated on the corpus callosum. (E) and (F) are the high magnification images of the highlighted regions of (C) and (D). The black arrow in (E) points to aggregated neurofilaments. Scale bar: 50 µm.

Activated microglial cells were particularly concentrated on corpus callosum (Figs. 3B and 3C), the largest cerebral white matter. To investigate whether microglial activation associates with white matter degeneration in corpus callosum, we conducted immunohistochemical staining of myelin basic proteins (MBP). There was no difference in the MBP staining between the left striatum transduced with scAAV8-hrGFP virus (Fig. S3A) and the uninjected right striatum (Fig. S3B). In contrast to the control scAAV8-hrGFP mice (Fig. 4A), MBP staining was decreased in the corpus callosum on the left hemisphere transduced with either scAAV8-D1shRNA1 (Fig. 4B) or scAAV8-D1shRNA3 (Fig. 4C). Under a higher magnification, localized swelling and disorganization of corpus callosum was observed in the left striatum transduced with scAAV8-D1shRNA1 (Fig. 4D) in comparison with the uninjected right striatum (Fig. 4E). In addition to corpus callosum, striatal white matter tracts were also degenerated in the left striatum (Fig. 4F) in contrast to the control right striatum (Fig. 4G). To investigate whether white matter degeneration may damage inside large-caliber axons, we conducted immunohistochemical staining of neurofilament light chains (NF-L), abundant structure proteins of large-caliber axons. Two adjacent paraffin sections were immunostained with anti-Iba-1 and anti-NF-L antibodies, respectively (Figs. 5A–5C). As expected, the left corpus callosum transduced with scAAV8-shRNA1 was swollen and disorganized with reduced NF-L staining in comparison with the control right corpus callosum (Figs. 5D and 5E). Striatal white matter tracts were also swollen and blebbed (Figs. 5F and 5G). None of these anatomical abnormalities were observed in mouse brains transduced with scAAV8-hrGFP virus (Fig. S4). Reduced NF-L staining was also observed in the corpus callosum transduced with scAAV8-D1shRNA3 virus (Fig. S5). Under a higher magnification, activated microglial cells were mostly co-localized with corpus callosum immunostained by anti-NF-L on an adjacent brain section (Figs. 6A–6D). Condensed aggregates of NF-L immunostaining were observed in both corpus callosum and striatal white matter tracts (Figs. 6C–6F), suggesting that D1shRNA-induced neuroinflammation caused demyelination and axon degeneration within white matter. We examined NF-L reduction in the corpus callosum of individual mice transduced with different scAAV8-D1shRNAs using anti-NF-L immunostaining as a marker (Table 2). NF-L reduction was arbitrarily ranked after within-section comparisons. None of the scAAV8-hrGFP mice displayed NF-L reduction in the injected brain hemisphere, whereas most mice injected with scAAV8-D1shRNA exhibited different degrees of NF-L reduction. Despite neuroinflammation and white matter degeneration, scAAV8-D1shRNA mice were indistinguishable from the control scAAV8-hrGFP mice in either locomotion or prepulse inhibition test (data not shown). No other behavioral tests were performed. Continuing deterioration of neuroinflammation and white matter degeneration may be required for the development of neurologic symptoms and behavioral abnormalities in scAAV8-D1shRNA mice. Given that shRNA immunogenicity may be a common “off-target” effect in mouse brains, gene silencing effects should be carefully interpreted in consideration of a potential involvement of neuroinflammation (Higa et al., 2017).

**Table 2:** NF-L immunostaining in corpus callosum.

	NF-L reduction in CC (IHC)					
	Negative	+ + +	+ +	+	No. of mice	Percentage of mice (%)
scAAV8-GFP	6	0	0	0	6	0
scAAV8-D1shRNA1	1	4	4	1	10	90
scAAV8-D1shRNA2	2	2	3	3	10	80
scAAV8-D1shRNA3	1	2	3	2	8	88

## Discussion

The scAAV8 virus, brain injuries from stereotaxic surgeries, and hrGFP over-expression cannot induce neuroinflammation in our studies. Over-expression of D1shRNAs causes neuroinflammation and white matter degeneration. Various factors, such as shRNA off-target silencing, Drd1 silencing, and shRNA immunogenicity, etc., may be involved in the development of neuroinflammation and white matter degeneration caused by scAAV8-D1shRNAs.

### shRNA off-target silencing

We designed three D1shRNAs that have different target sequences. No common potential off-target genes were found among these three D1shRNAs by *in silico* analyses. Therefore, it is unlikely that D1shRNA off-target silencing effects play a major role in induction of neuroinflammation. We did not include a nonspecific shRNA as a negative control to assess D1shRNA off-target silencing effects since different shRNAs have different sets of off-target genes.

### Drd1 silencing

The limitation of our studies is that we cannot conclude whether Drd1 silencing is involved in the development of neuroinflammation due to lack of nonspecific shRNA (scrambled shRNAs) controls. However, McBride et al. (2008) reported that over-expression of nonspecific shRNAs is sufficient to activate microglial cells in mouse striatum. Importantly, not all shRNAs can induce microglial activation (McBride et al., 2008), suggesting that shRNAs have sequence-dependent but not sequence-specific effects on activating innate immune responses. Therefore, it is difficult to use nonspecific shRNAs to rule out a potential role of Drd1 silencing in activation of the innate immune system. Since Drd1 can be induced in activated microglial cells (Mastroeni et al., 2009), and negatively regulates the NLRP3 inflammasomes to prevent excessive inflammation (Yan et al., 2015), it is possible that Drd1 silencing may contribute to extensive neuroinflammation induced by scAAV8-D1shRNAs. The role of Drd1 in neuroinflammation may be investigated in the future by injecting scAAV8-D1shRNA virus into *Drd1* knockout mouse brains.

### shRNA immunogenicity

It is likely that D1shRNA immunogenicity plays an important role in microglial activation and white matter degeneration, as indicted by McBride et al. (2008). siRNAs/shRNAs have been well studied for eliciting innate immune responses by activating Toll-like receptor 3 (TLR-3) and/or protein kinase RNA-activated (PKR) (Grimm et al., 2006; Kariko et al., 2004; Sledz et al., 2003). TLR3 is activated by dsRNAs with a length of at least 40 to 50 bp (Liu et al., 2008). The dsRNA stems of D1shRNA1, D1shRNA2, and D1shRNA3 are between 21 to 23 bp that are insufficient to dimerize and activate TLR-3. PKR is localized in cytoplasm and can be activated by dsRNAs with a length of at least 30 bp (Lemaire et al., 2008). However, shorter dsRNAs were reported to bind and activate PKR (Mayo et al., 2016; Puthenveetil et al., 2006; Sledz et al., 2003). Therefore, D1shRNAs may have activated PKR in neurons and/or glial cells to initiate inflammatory responses. Sequence-dependent TLR7 was reported to mediate siRNA immunogenicity (Hornung et al., 2005). TLR7 may be another candidate downstream effector of D1shRNAs in the activation of CNS innate immune responses.

### Innate immune cells

AAV8 virus transduces both neuronal cells and glial cells (Watakabe et al., 2015). Transduction of glial cells, particularly microglial cells by scAAV8-D1shRNAs, may contribute to extensive neuroinflammation and white matter degeneration seen in our studies. We found that activated microglial cells were predominantly concentrated on corpus callosum and co-localized with demyelination, suggesting that the activated microglial cells were polarized to the M1 cytotoxic phenotype (Orihuela, McPherson & Harry, 2016). Activated microglial cells release excessive glutamate (Barger et al., 2007). Oligodendrocytes are the most vulnerable CNS cells to glutamate excitotoxicity (McDonald et al., 1998). Oligodendrocytes in scAAV8-D1shRNA mice may have been damaged by excessive glutamate and thereby were attacked by activated microglial cells. Since peripheral immune cells infiltrated into inflammatory brain tissue in scAAV8-D1shRNA mice, we cannot determine whether activated microglial cells were derived from infiltrated peripheral macrophages or CNS resident microglial cells. Nevertheless, CNS resident microglial cells are likely one of the early key players activated by D1shRNA over-expression, which subsequently causes neuroinflammation to breakdown blood brain barrier for infiltration of peripheral immune cells including macrophages. Interactions between activated CNS innate immune cells and infiltrated peripheral adaptive immune cells exacerbate white matter degeneration.

### RNA virus and retroviral RNA in MS

CNS demyelination can be naturally induced by a variety of virus in animals including visna virus, caprine arthritis-encephalitis virus, Theiler’s murine encephalomyelitis virus, Semliki Forest virus, etc (Pachner, 2011). All these are RNA viruses that produce massive viral dsRNAs to activate the innate immune system in infected host cells, suggesting that human RNA viruses are candidate pathogens in some human MS patients. Recent studies demonstrated that massive dsRNA can also be produced in the absence of virus infection by transcription of repetitive genomic sequences or endogenous retroviral sequences of the genome due to aberrant epigenetic modifications, which subsequently activates the innate immune system to over-express pro-inflammatory cytokines (Chiappinelli et al., 2015; Leonova et al., 2013; Roulois et al., 2015). McBride et al. (2008) and our studies found that chronic massive production of dsRNA/shRNA induces neuroinflammation and white matter degeneration, suggesting that epigenetic aberration may be involved in the pathogenesis of human MS and other inflammatory diseases.

##  Supplemental Information

10.7717/peerj.3905/supp-1 Figure S1 Design of D1shRNAs and suppression of Drd1 protein in mouse striatum (A) Three different shRNAs were designed to target different sites of mouse Drd1 gene. Immunohistochemical staining of Drd1 was performed using rabbit anti-Drd1 antibody in mouse striatum transduced with either scAAV8-hrGFP (B) or scAAV8-D1shRNA1 (C) virus. The virus was injected into the left striatum only. Reduced Drd1 protein was observed in the left striatum injected with scAAV8-D1shRNA1 virus, but not with scAAV8-hrGFP virus. High magnification of Drd1 reduction was shown in the left striatum transduced with scAAV8-D1sdhRNA1 virus (D) in comparison with Drd1 expression in the right control striatum (E). Suppression of Drd1protein expression was also observed using scAAV8-D1shRNA2 (F) and scAAV8-D1shRNA3 (H) in comparison with Drd1 expression in their right control striatum (G) and (I). Scale bar: 50 µm.

10.7717/peerj.3905/supp-2 Figure S2 Mild neuroinflammation in striatum transduced with scAAV8-D1shRNA2 virus (A) Immunohistochemical staining of Iba-1 in mice injected with scAAV8-D1shRNA2 virus. Microglial cells were activated to surrounding blood capillaries in the left striatum transduced with the scAAV8-D1shRNA2 virus. (B) and (C) are the higher magnifications of mild neuroinflammation around the blood capillaries. Scale bar: 50 µm.

10.7717/peerj.3905/supp-3 Figure S3 Immunohistochemical staining of MBP There is no difference in MBP staining in the left corpus callosum transduced with scAAV8-hrGFP virus (A) in comparison with the control right corpus callosum without virus injection (B). Scale bar: 100 µm. MBP was reduced (black arrow) on the left corpus callosum (C) of mice injected with scAAV8-D1shRNA3 in comparison with the normal right corpus callosum (D). Scale bar: 200 µm. Under a higher magnification, MBP staining was also decreased in striatal white matter tracts that were swollen and blebbed (black arrowheads) in the left striatum transduced with scAAV8-D1shRNA3 (E) in comparison with normal white matter tracts in the control right striatum (F). Scale bar: 60 µm.

10.7717/peerj.3905/supp-4 Figure S4 Absence of neuroinflammation and white matter degeneration in the left striatum transduced with scAAV8-hrGFP virus Two consecutive paraffin sections from mice injected with scAAV8-hrGFP virus were immunostained 7 weeks post-injection with either anti-Iba-1 (A) or anti-NF-L (B) antibody. There was no activation of microglial cells in the left striatum transduced with scAAV8-hrGFP virus in comparison with the uninjected control right striatum. There was no reduction of NF-L staining in the corpus callosum on the left side (C) transduced with the virus in comparison with the control right side without virus infection (D). Scale bar: 150 µm.

10.7717/peerj.3905/supp-5 Figure S5 White matter degeneration and neurofilament reduction in striatum transduced with scAAV8-D1shRNA3 virus Two consecutive brain paraffin sections from mice injected with scAAV8-D1shRNA3 virus were immunostained with either anti-Iba-1 (A) or anti-NF-L (B) antibody. Extensive microglial activation as shown by anti-Iba-1 immunostaining was observed in the left striatum transduced with the scAAV8-D1shRNA3 virus in comparison with the control right striatum. Decreased NF-L staining was observed in the left corpus callosum (C) compared with the control right corpus callosum (D). Scale bar: 200 µm. Neurofilament staining was reduced in blebbed striatal white matter tracts (black arrow) in the left striatum transduced with the virus (E) in contrast to the control right striatum (F). Scale bar: 60 µm.

10.7717/peerj.3905/supp-6 Data S1 Western blot raw data with mouse anti-Drd1

10.7717/peerj.3905/supp-7 Data S2 Western blot raw data using anti-mouse IgG

10.7717/peerj.3905/supp-8 Data S3 Western blot raw data mouse IgG week 4 to 6

10.7717/peerj.3905/supp-9 Data S4 Western blot raw data mouse IgG week 7 to 15 Week 7, 8, 9, 15.

10.7717/peerj.3905/supp-10 Data S5 Western blot raw data NR1 week 4 to 6 Normalization controls.

10.7717/peerj.3905/supp-11 Data S6 Western blot raw data NR1 week 7 to 15 Normalization controls for week 7, 8, 9, and 15.

10.7717/peerj.3905/supp-12 Table S1 IgG immunostaining in individual mouse striatum

## Additional Information and Declarations

### Data Availability

The following information was supplied regarding data availability: The raw Western blot data is found in Figs. 1 and 2.
