# Supplementary figures and images for: A novel animal model for neuroinflammation and white matter degeneration
Source: PeerJ. 2017 Oct 31;5:e3905. doi: 10.7717/peerj.3905 (PMC5669272; doi:10.7717/peerj.3905)

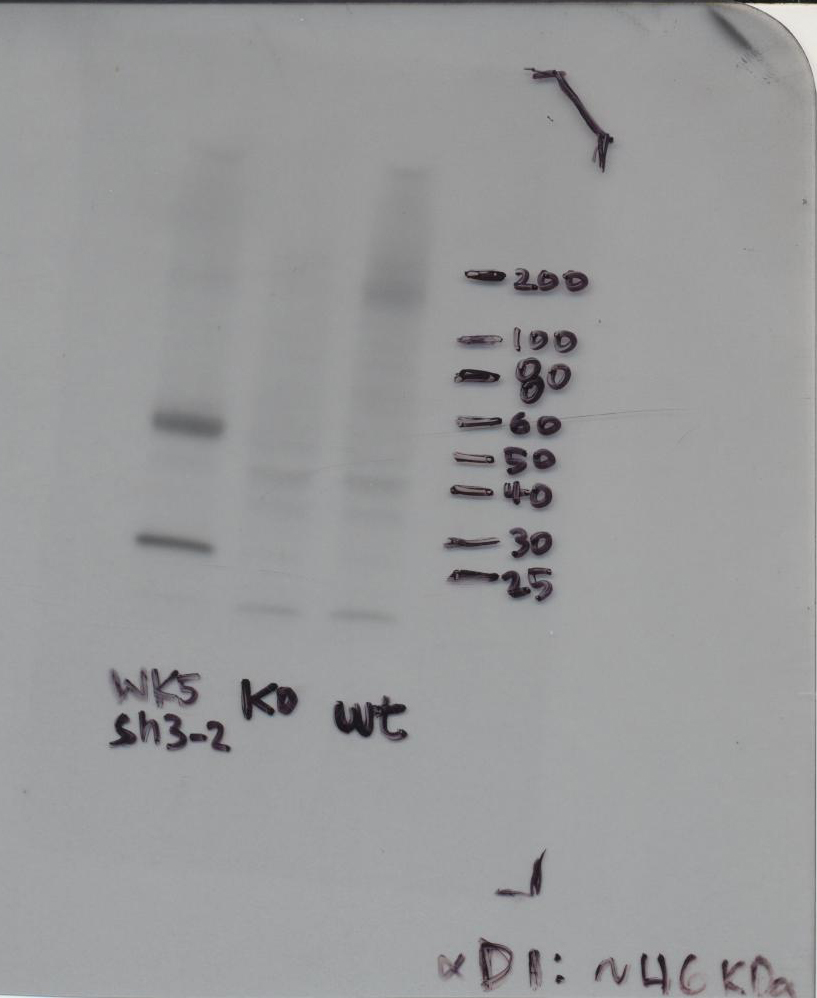

Supplement: Data S1 [file peerj-05-3905-s006.jpg]

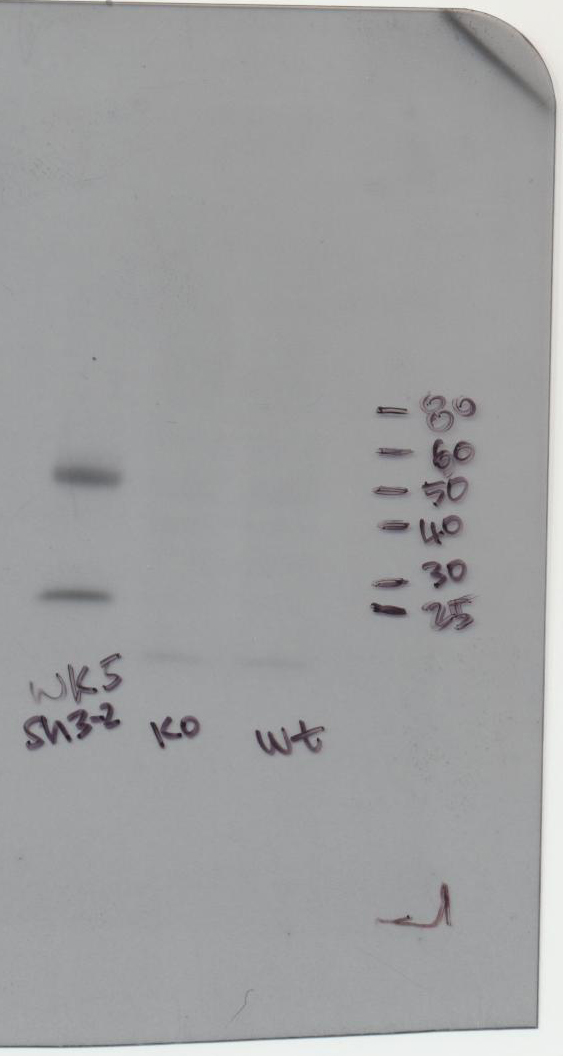

Supplement: Data S2 [file peerj-05-3905-s007.jpg]

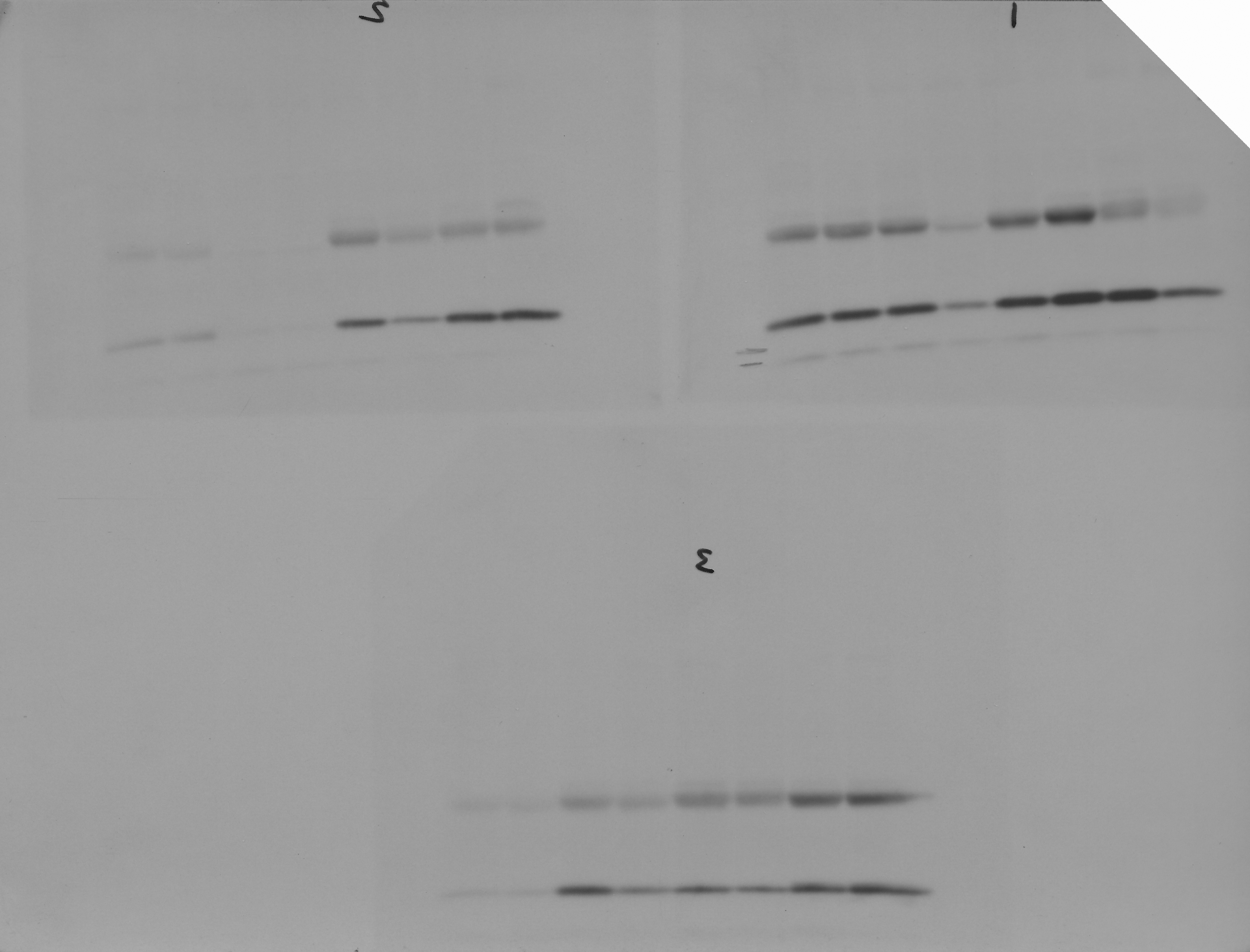

Supplement: Data S3 [file peerj-05-3905-s008.jpg]

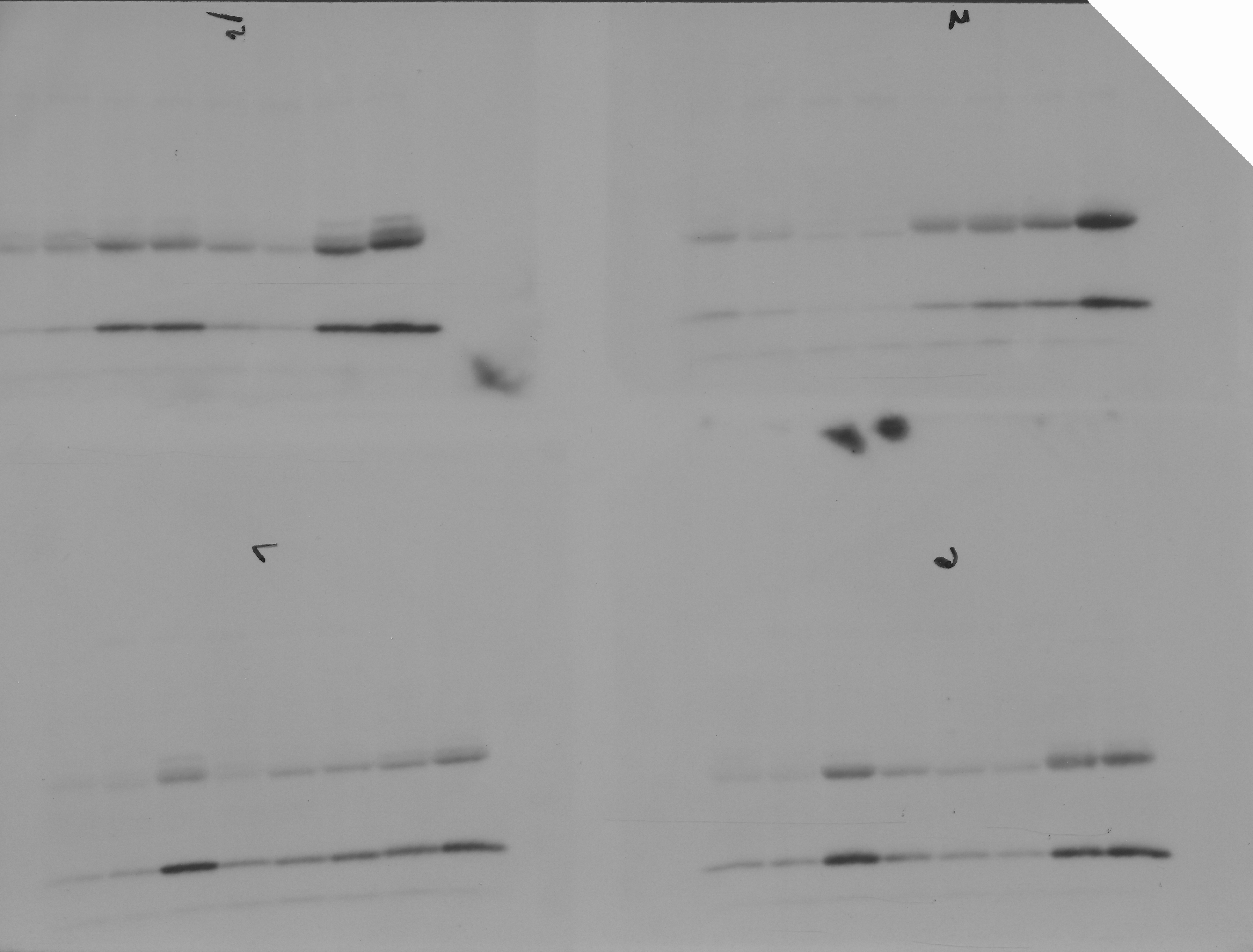

Supplement: Data S4 — Week 7, 8, 9, 15. [file peerj-05-3905-s009.jpg]

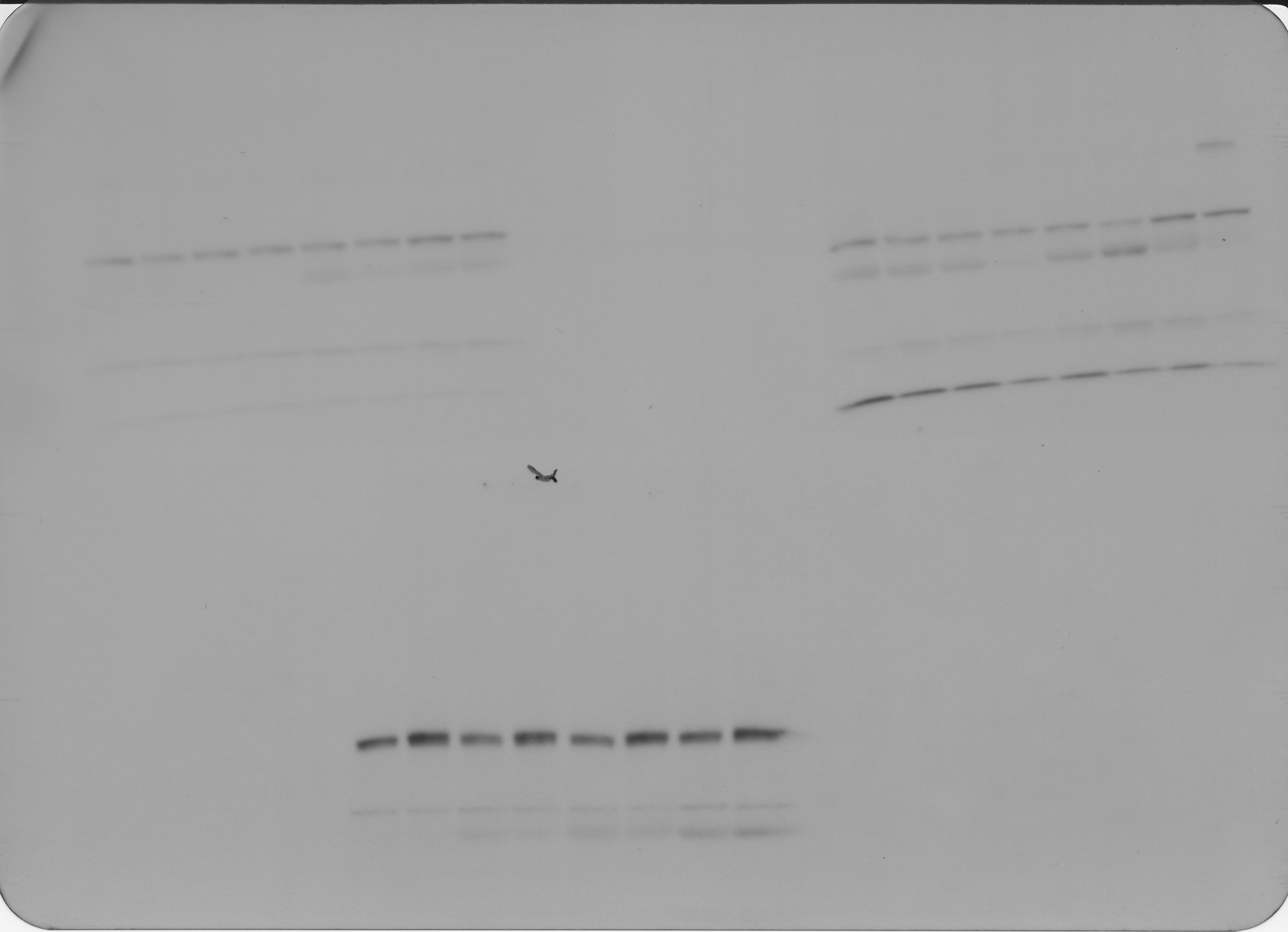

Supplement: Data S5 — Normalization controls. [file peerj-05-3905-s010.jpg]

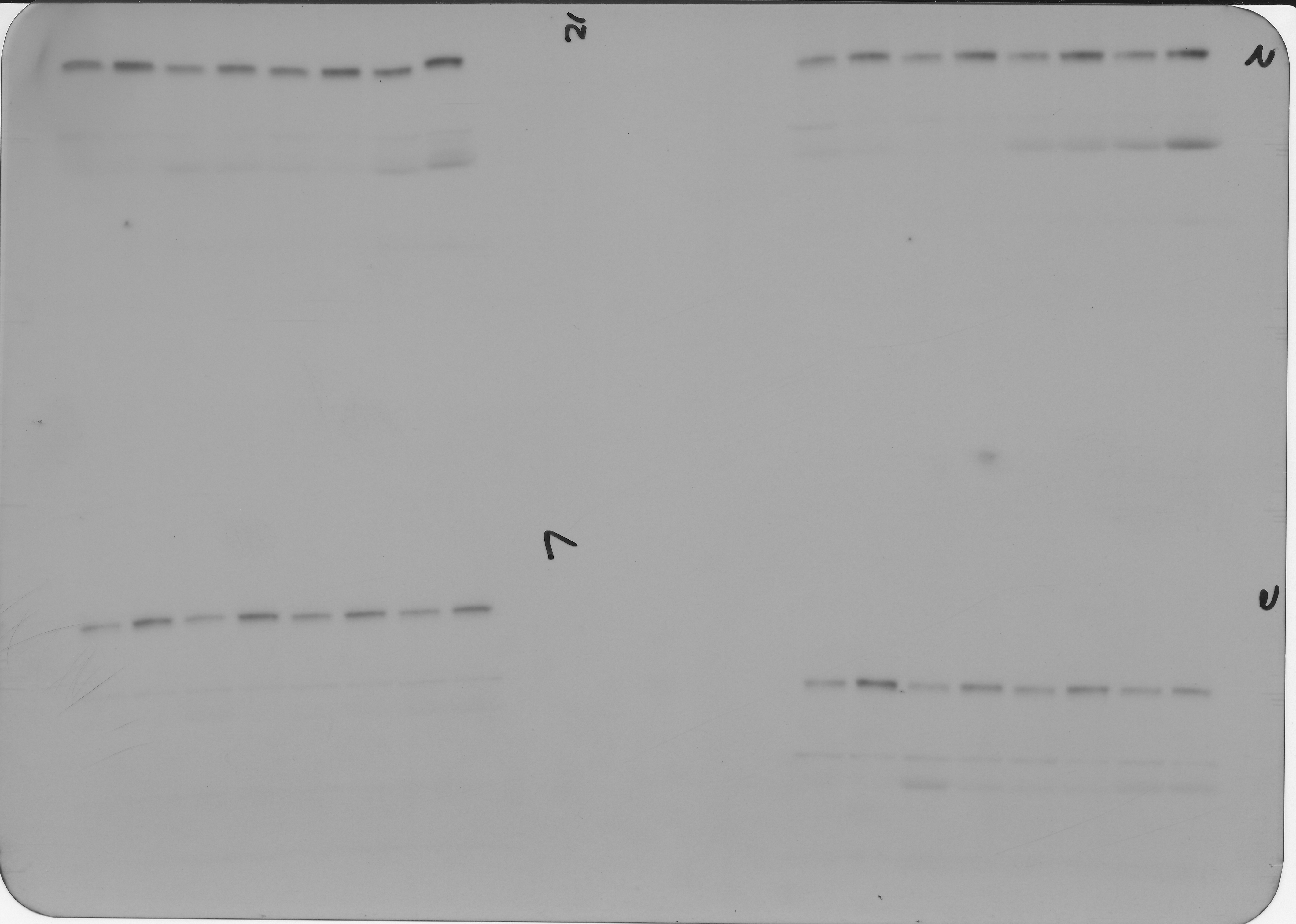

Supplement: Data S6 — Normalization controls for week 7, 8, 9, and 15. [file peerj-05-3905-s011.jpg]
