# Supplementary material for: A novel animal model for neuroinflammation and white matter degeneration
Source: PeerJ. 2017 Oct 31;5:e3905. doi: 10.7717/peerj.3905 (PMC5669272; doi:10.7717/peerj.3905)
Supplement: Table S1 [file peerj-05-3905-s012.docx]

|  |  |  | Mouse IgG Intensities (IHC) | | | | |
| --- | --- | --- | --- | --- | --- | --- | --- |
|  |  | Negative | +++ | ++ | + | No. of  mice | Percentage of mice (%) |
| scAAV8-GFP | | 6 | 0 | 0 | 0 | 6 | 0 |
| scAAV8-D1shRNA1 | | 0 | 6 | 3 | 1 | 10 | 100 |
| scAAV8-D1shRNA2 | | 1 | 2 | 4 | 3 | 10 | 90 |
| scAAV8-D1shRNA3 | | 1 | 3 | 2 | 2 | 8 | 88 |

**Supplemental Table I. IgG Immunostaining in Individual Mouse Striatum**
